# Genomic and physiological properties of *Anoxybacterium hadale* gen. nov. sp. nov. reveal the important role of dissolved organic sulfur in microbial metabolism in hadal ecosystems

**DOI:** 10.3389/fmicb.2024.1423245

**Published:** 2024-08-16

**Authors:** Junwei Cao, Baoying Shao, Jing Lin, Jie Liu, Yiran Cui, Jiahua Wang, Jiasong Fang

**Affiliations:** ^1^College of Oceanography and Ecological Science, Shanghai Ocean University, Shanghai, China; ^2^Laboratory for Marine Biology and Biotechnology, Qingdao National Laboratory for Marine Science and Technology, Qingdao, China

**Keywords:** hadal environment, Clostridia bacterium, organic sulfur, cysteine, uncultured microbe

## Abstract

Hadal zones account for the deepest 45% of the oceanic depth range and play an important role in ocean biogeochemical cycles. As the least-explored aquatic habitat on earth, hadal ecosystems contain a vast diversity of so far uncultured microorganisms that cannot be grown on conventional laboratory culture media. Therefore, it has been difficult to gain a true understanding of the detailed metabolic characteristics and ecological functions of those difficult-to-culture microorganisms in hadal environments. In this study, a novel anaerobic bacterial strain, MT110^T^, was isolated from a hadal sediment–water interface sample of the Mariana Trench at 10,890 m. The level of 16S rRNA gene sequence similarity and percentage of conserved proteins between strain MT110^T^ and the closest relatives, *Anaerovorax odorimutans* DSM 5092^T^ (94.9 and 46.6%) and *Aminipila butyrica* DSM 103574^T^ (94.4 and 46.7%), indicated that strain MT110^T^ exhibits sufficient molecular differences for genus-level delineation. Phylogenetic analyses based on both 16S rRNA gene and genome sequences showed that strain MT110^T^ formed an independent monophyletic branch within the family Anaerovoracaceae. The combined evidence showed that strain MT110^T^ represents a novel species of a novel genus, proposed as *Anoxybacterium hadale* gen. nov. sp. nov. (type strain MT110^T^ = KCTC 15922^T^ = MCCC 1K04061^T^), which represents a previously uncultured lineage of the class Clostridia. Physiologically, no tested organic matter could be used as sole carbon source by strain MT110^T^. Genomic analysis showed that MT110^T^ had the potential capacity of utilizing various carbon sources, but the pathways of sulfur reduction were largely incomplete. Our experiments further revealed that cysteine is one of the essential nutrients for the survival of strain MT110^T^, and cannot be replaced by sulfite, leucine, or taurine. This result suggests that organic sulfur compounds might play an important role in metabolism and growth of the family Anaerovoracaceae and could be one of the key factors affecting the cultivation of the uncultured microbes. Our study brings a new perspective to the role of dissolved organic sulfur in hadal ecosystems and also provides valuable information for optimizing the conditions of isolating related microbial taxa from the hadal environment.

## 1 Introduction

Hadal zones at water depths below 6,000 m account for the deepest 45% of the oceanic depth range and host active and diverse biological communities, showing a great significance in the ocean ecosystem. Comprised primarily of troughs and trenches with elevated hydrostatic pressure and hydrotopographically isolated nature, the hadal oceans are the least-explored aquatic biosphere on Earth (Nunoura et al., 2015; Liu et al., 2018). Mariana Trench sediments are subject to extreme physical and chemical conditions, with high pressure up to 1,100 atmosphere and low temperature near freezing. Additionally, the lack of sunlight and limited organic matter availability contribute to reduced oxygen levels in the sediment (Glud, 2008; Liu et al., 2018). The organic matter in the sediment is mainly composed of dead and decaying marine organisms, such as phytoplankton and marine snow, which fall to the bottom of the trench. These organic compounds are rich in sulfur-containing compounds, including amino acids, peptides, nucleotides, and lipids, leading to the abundance of sulfur in the hadal seawater and sediments (Danovaro et al., 2002; Jamieson et al., 2010; Guan et al., 2019). The sulfur-containing amino acids, cysteine (Cys) and methionine (Met), are particularly abundant and can support the growth of a diverse and specialized microbial community in this extreme environment, including both heterotrophic bacteria and subsequent sulfur-oxidizing bacteria, which play an important role in the biogeochemical cycling of sulfur (Hu et al., 2018).

Bacillota (‘Firmicutes’), currently containing seven classes, “Bacilli,” “Clostridia,” “Culicoidibacteria,” “Limnochordia,” “Negativicutes,” “Thermolithobacteria,” and “Erysipelotrichia,” includes bacteria with highly diverse phenotypic and genetic characteristics and are prevalent in various environments, including the Mariana Trench water column and sediment (Li et al., 2019; Neupane et al., 2020; Liu et al., 2023). Some members of the Bacillota phylum are capable of degrading complex organic compounds, such as lignin and cellulose, which are normally refractory, or difficult to break down (Qin, 2016). In particular, a group of Bacillota, known as the Clostridia, has been found to be abundant (12.75% of the community) in the Mariana Trench sediment and was thought to contribute significantly to the overall metabolic activity of the microbial community in this environment (Li et al., 2019). Some of these Clostridia are capable of producing hydrogen and acetate, which can serve as important energy sources for other microbes in the sediment (Matthies et al., 2000; Kim et al., 2016).

Although the two Clostridia genera, *Anaerovorax* and *Aminipila*, have been reported for over 24 years and are widely distributed in many environments (Liu et al., 2011; Staudinger et al., 2011; Kobayashi et al., 2013; Tischer et al., 2013; Li et al., 2014; Aromokeye et al., 2020; Kim et al., 2021; Wei et al., 2021; Hyunwoo et al., 2023), they currently consist of merely four cultured species (Matthies et al., 2000; Ueki et al., 2018). *Anaerovorax*, a Clostridia genus proposed by Matthies et al. (2000), comprises only one known species, the type species *Anaerovorax odorimutans.*^1^ *Anaerovorax odorimutans* is an anaerobic, Gram-positive, non-spore-forming bacterium that only utilize putrescine, 4-aminobutyrate and 4-hydroxybutyrate as growth substrates (Matthies et al., 2000). The genus *Aminipila* was proposed by Ueki et al. (2018) and belongs to the class Clostridia in the phylum Bacillota (‘Firmicutes’). Currently, there are only three species of the genus with validly published names (see Footnote 1). *Aminipila* members are aerotolerant or obligately anaerobic, non-spore-forming bacteria that do not utilize carbohydrates (Wei et al., 2021). Moreover, previous study showed that these isolates were unable to grow with more than 30 different substrates tested, such as sugars, organic acids, alcohols, amino acids and other amines (Matthies et al., 2000; Wei et al., 2021). Therefore, the metabolic properties and ecological functions of these microbes are largely unknown.

In this study, we isolated a novel anaerobic Clostridia strain, MT110^T^, from a sample taken at the sediment–water interface of the Mariana Trench (−10,890 m). Strain MT110^T^ is phylogenetically related to species within the genera *Anaerovorax* and *Aminipila*, and it also showed negative growth when tested with various single substrates as the sole carbon source. Here, we present the unique phylogenetic position of strain MT110^T^, along with a comparative genomic analysis, and demonstrate its carbon and sulfur metabolic characteristics through the reconstruction of metabolic pathways and comprehensive biological experiments. Our findings provide insights into the trophic strategies of the hadal microbiome and offer valuable references for optimizing isolation conditions for related taxa.

## 2 Materials and methods

### 2.1 Strains and culture conditions

A water sample was collected in the sediment–water interface at a depth of 10,890 m from the Mariana Trench (142.4°E, 11.4°N; station MT) in December 2016, during the MT2016 cruise of the research vessel “*Zhangjian*.” The sample was collected using Niskin bottles fitted on a Lander (Shanghai Ocean University, China) and anaerobically preserved in sealed sterile vials at 4°C onboard. One subsample was used to inoculate a 1:10 strength TRM medium (Cao et al., 2016), prepared with a gas phase of N_2_ (200 kPa) and incubated anaerobically at 28°C. After 10 days of incubation, the enrichments were subcultured under the same conditions, and purified by eight repeated dilution-to-extinction series. One isolate, designated MT110^T^, was obtained. Stock cultures were stored at −80°C with 5% (v/v) DMSO. The phylogenetically related type strains, *Anaerovorax odorimutans* DSM 5092^T^ (=NorPut^T^) (Matthies et al., 2000) and *Aminipila butyrica* DSM 103574^T^ (=FH042^T^) (Ueki et al., 2018) were obtained from the Leibniz Institute DSMZ–German Collection of Microorganisms and Cell Cultures (DSMZ). The routine cultivation of the strains and most phenotypic tests were carried out on M89 medium, unless noted otherwise. M89 medium contained (L^−1^) 700 mL seawater, 300 mL distilled water, 1 g tryptone, 2 g yeast extract, 2 g casamino acid, 2 g 4-aminobutyric acid, 3 g HEPES buffer, 0.25 g L-cysteine hydrochloride dihydrate (as a reducing agent), 1 mg sodium resazurin (as a redox indicator). The pH of the medium was adjusted to 7.0 with 5 M NaOH at room temperature. After autoclaving, 1 mL of vitamin solution (Balch et al., 1979) and 1 mL of selenite-tungstate solution (Widdel and Bak, 1992) were added.

### 2.2 Phenotypic analyses

Unless noted otherwise, physiological tests were carried out anaerobically in M89 medium, in duplicate. Morphological characteristics of strain MT110^T^ grown on M89 medium at 28°C for 90 h were observed by using light microscopy (Olympus BX60 and CX40) and scanning electron microscopy (FEI Quanta 200). Spore formation was assessed by observation of cells after Gram-staining as well as by means of phase-contrast microscopy. Determination of the temperature range for growth was carried out at 4, 10, 15, 20, 28, 35, 42, and 45°C. Salt tolerance was tested at 28°C with various concentrations of NaCl (0, 0.5, 1.0, 2.0, 3.0, 4.0, 5.0, and 6.0%, w/v). The pH range for growth was tested from pH 4.0 to 11.0 (initial pH at 20°C) with increments of 0.5 unit.

Utilization of substrate as sole carbon source was tested in BM liquid medium with each substrate added at 20 mM as the final concentration. BM medium contained 1,000 mL pure water, 5 g NaCl, 0.7 g KCl, 0.1 g CaCl_2_·2H_2_O, 2 g Na_2_SO_4_, 2 g MgCl_2_·6H_2_O, 3 g HEPES, 0.33 g NH_4_Cl, 0.25 g Na_2_S·9H_2_O (as a reducing agent). The pH of the medium was adjusted to 7.5. After autoclaving, 1 mL of vitamin solution, 1 mL of selenite-tungstate solution, 1 mL trace element solution, 1 mL 5% K_2_HPO_4_ and 1 mL 5% KH_2_PO_4_ were added. Growth was determined by OD_600_ of culture. All tests were carried out in duplicate.

As no sole substrate was found to be used as sole carbon source to support the growth of strain MT110^T^, utilization of carbohydrates was tested via supplement of additional substrates (glucose, fructose, ribose, inositol, xylose, sucrose, cellodisaccharide, and water-soluble starch) into BMY liquid medium (BM medium with 2 g/L yeast extracts). The final concentration of each carbohydrate was 20 mM, except for starch (5 g/L).

Utilization of sulfur-containing methionine and/or cysteine was tested in BM18A liquid medium supplemented with cysteine and/or methionine. BM18A medium was prepared with BM medium by adding of 18 amino acids (alanine, arginine, asparagine, aspartic acid, glutamine, glutamic acid, glycine, histidine, isoleucine, leucine, lysine, phenylalanine, proline, serine, threonine, tryptophan, tyrosine, and valine). Each amino acid was added at 0.02% (w/v) as the final concentration. The reducing agent Na_2_S·9H_2_O was added at two levels, 0.05 and 0.025% (w/v) as the final concentration, respectively.

### 2.3 Chemotaxonomic analysis

Chemotaxonomic analyses were performed on mid-to late-exponential-phase of growth cultures grown for 90 h on M89 medium. The cellular fatty acids in whole cells were saponified, methylated and extracted using the standard protocol of MIDI (Sherlock Microbial Identification System, version 6.0B). The cellular fatty acids were analyzed by GC (Agilent Technologies 6,850) and identified by using the TSBA6.0 database of the Microbial Identification System (Sasser, 1990). The fatty acid and polar lipid profiles of reference strains *Anaerovorax odorimutans* DSM 5092^T^ and *Aminipila butyrica* DSM 103574^T^ were performed in parallel with strain MT110^T^ under the same condition.

### 2.4 Phylogenetic and phylogenomic analyses

Genomic DNA was extracted with the QIAGEN QiAamp DNA mini kit (QIAGEN, Düsseldorf, Germany) following the manufacturer’s standard protocol. The 16S rRNA gene was sequenced by Sanger method using the primers Bac8F (5′-AGA GTT TGA TCA TGG CTC AG-3′), and U1492R (5′-GGT TAC CTT GTT ACG ACT T-3′) (Liu et al., 2020). The 16S rRNA gene sequence of strain MT110^T^ was deposited in the GenBank database under the accession number OQ442995. Pairwise 16S rRNA gene sequence similarity was determined using the EzBioCloud server^2^ (Yoon et al., 2017). Phylogenetic analysis of 16S rRNA gene was performed using the software MEGA X (Kumar et al., 2018). Distances were calculated using the Kimura two-parameters model and clustering was performed with the maximum-likelihood (Felsenstein, 1981) algorithm. The robustness of the inferred topology was calculated by bootstrap analysis based on 1,000 replications.

The complete genome of stain MT110^T^ was sequenced using Oxford Nanopore Technology (Nextomics Biosciences Co., Ltd., Wuhan, China). The genome was assembled using Canu (v1.5) (Koren et al., 2017) and further polished using nanopolish (v0.8.3) (Loman et al., 2015), using default parameters. The completeness and contamination of the assembled genome were accessed using CheckM2 (Chklovski et al., 2023). Gene annotations were performed by the NCBI prokaryotic genome annotation pipeline (PGAP) (Tatusova et al., 2016). The complete genome sequence of strain MT110^T^ was deposited in the GenBank database under the accession number CP042469. The 120 conserved bacterial marker genes of the GTDB taxonomy were used to study the phylogeny of strain MT110^T^ and its related strains. The sequences of 120 concentrated proteins in the genomes were predicted using GTDB-Tk (database version: Release 07-RS207) (Chaumeil et al., 2019) and separately aligned using Clustal Omega (Sievers and Higgins, 2021). All aligned sequences were manually degapped and tandemly connected (5,038 amino acid residue in total after degap). The phylogenetic tree was constructed using FastTree2 with the neighbor-joining method (Price et al., 2010), and a bootstrap analysis with 1,000 replicates was performed to assess the robustness of the tree. Finally, the phylogenetic tree was plotted using iTOL (Letunic and Bork, 2021). The percentage of conserved proteins (POCP) between two genomes was calculated according to Qin et al. (2014).

### 2.5 Gene annotation and metabolic pathway reconstruction

The NCBI prokaryotic genome annotation pipeline was used in ORF prediction and gene annotation (Tatusova et al., 2016). Gene functional categories were identified by a BLASTp search in the Clusters of Orthologous Groups (COG) database (Tatusov et al., 2001). Metabolic pathway analysis was performed by searching the KEGG GENES database with BlastKOALA (Kanehisa et al., 2016). The genomic islands were predicted using IslandViewer 4 (Bertelli et al., 2017). The transporters were predicted by BLASTp against TransportDB 2.0 (Elbourne et al., 2017).

### 2.6 Genomic comparison

Protein families of strain MT110^T^ and phylogenetically related strains were clustered using a local OrthoMCL 2.0.9 (Li et al., 2003) with the following cutoff values: identity, 50%; query coverage, 50%; *E-*value, 1e-10; score, 40; and MCL Markov clustering inflation index, 1.5. The protein families employed by only one strain were considered as strain-specific.

## 3 Results

### 3.1 Strain isolation and description

A novel anaerobic bacterium, MT110^T^, was isolated by the extinction dilution technique from a hadal sediment–water interface sample (−10,890 m) of the Mariana Trench. Purity of the culture was verified by microscopy and further confirmed by 16S rRNA gene sequencing.

Strain MT110^T^ is a strictly anaerobic, Gram-positive bacterium consisting of straight to gently curved rods (length: 2.4–3.4 μm, diameter: 0.4–0.6 μm, Supplementary Figure S1). The bacterium typically occurs singly. Spores were not observed in cells cultured in M89 medium by microscopy. It can grow in a range of NaCl concentrations from 0 to 5% (optimum: 0.5%), at pH levels from 6.0 to 9.0 (optimum: 7.5–8.0), and at temperatures ranging from 10 to 42°C (optimum: 28°C). The main fatty acids present in strain MT110^T^ are C14:0 (47.7%), C16:1 ω7c/ω6c (Summed feature 3, 10.7%), C16:0 (10.3%), and C18:1 ω9c (10.0%). These fatty acid profiles noticeably differ from those of the two reference strains (Table 1). C_16:1_ *ω*7*c*/*ω*6*c* (Summed feature 3), C16:1 *ω*9*c* and C_14:0_ iso was present a higher amount in strain MT110^T^ than *Anaerovorax odorimutans* DSM 5092^T^ and *Aminipila butyrica* DSM 103574^T^. Reversely, strain MT110^T^ contained a lower amount of C_16:0_, C_17:1_ iso I/anteiso B (Summed feature 4) and C_19:1_ iso I than *Anaerovorax odorimutans* DSM 5092^T^ and *Aminipila butyrica* DSM 103574^T^.

**Table 1:** Differentiating characteristics between strain MT110^T^ and reference strains.

Characteristic	1	2	3
Isolation source	Hadal sediment–water interface	Methanogenic reactor of cattle farms	Brackish water sediment
Cell size	0.4–0.6 × 2.4–3.4	0.7–1.0 × 3.0–8.0	0.7–0.8 × 1.9–2.7
Temperature range (optimum)	10–42 (28)	10–35 (30)	12–50 (37)
pH range (optimum)	6.0–9.0 (7.5–8.0)	6.0–8.5 (6.5)	5.1–8.0 (7.2–7.6)
NaCl tolerance range (g l^−1^) (optimum)	0–5 (0.5)	0–20 (0)	0–20 (NA)
4-aminobutyric acid	−	−	+
Casamino acids	−	+	−
G+C content (mol%)	44.6	44.7	31.5
Fatty acids			
C_12:0_	2.3	7.9	0.6
C_16:1_ *ω*9*c*	8.5	ND	ND
C_14:0_	47.7	32.5	48.6
C_14:0_ iso	1.0	ND	ND
Sum In Feature 3 (C_16:1_ *ω*7*c*/*ω*6*c*)	10.7	8.9	0.7
C_16:0_	10.3	17.1	14.7
C_17:1_ iso *ω*10*c*	ND	ND	0.5
Sum In Feature 4 (C_17:1_ iso I/anteiso B)	2.6	7.5	14.0
C_18:1_ *ω*9*c*	10.0	11.0	1.9
C_19:1_ iso I	0.5	2.4	3.1

Strains: 1, MT110^T^; 2, *Aminipila butyrica* DSM 103574^T^; 3, *Anaerovorax odorimutans* DSM 5092^T^.+, Positive; −, negative; NA, not available; ND, not detected.

### 3.2 Phylogenetic and phylogenomic analyses

**Figure 1:**
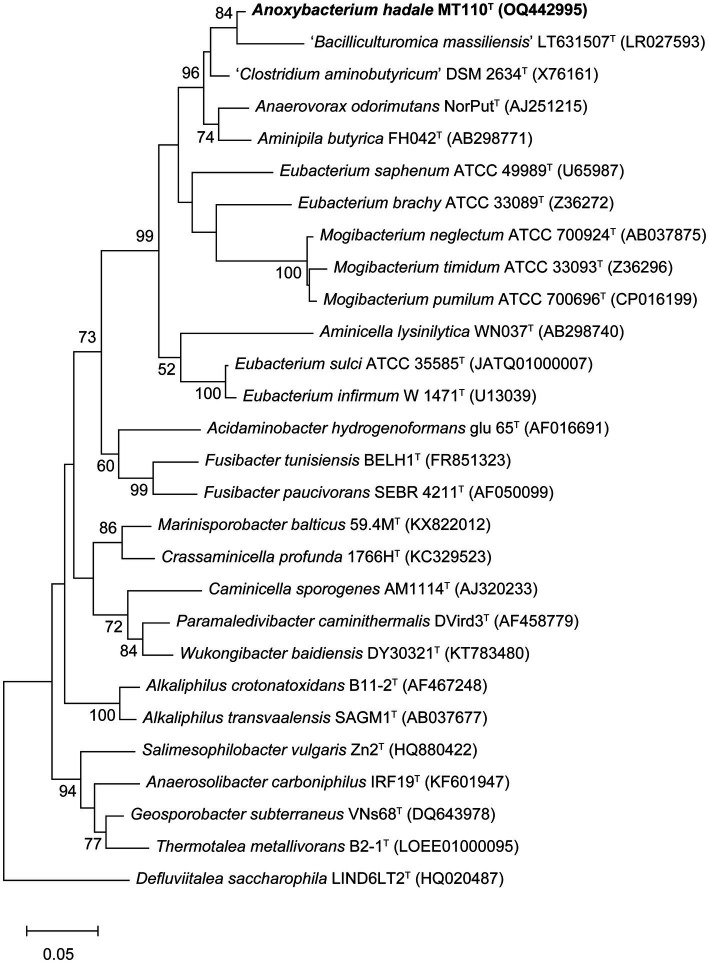
Maximum-likelihood tree showing the phylogenetic positions of strain MT110^T^ and related species, based on 16S rRNA gene sequences. Bootstrap values ≥50% (expressed as percentages of 1,000 replications) are shown at branch nodes. Bar, 0.05 nucleotide substitution rate (K_nuc_) units. *Defluviitalea saccharophila* LIND6LT2^T^ (HQ020487) was used as outgroup.

Almost a full-length 16S rRNA gene sequence (1,472 bp) of strain MT110^T^ was determined. The highest sequence similarity of 94.9% was obtained with *Anaerovorax odorimutans* DSM 5092^T^, followed by *Aminipila butyrica* DSM 103574^T^ with 94.4% similarity. The level of 16S rRNA gene sequence similarity indicated that strain MT110^T^ exhibits sufficient molecular differences for genus-level delineation, as it falls below the recommended threshold value of 95% for differentiating between genera (Johnson et al., 2019). Based on the 16S rRNA gene phylogenetic analysis (Figure 1), the novel isolate was clearly placed in a distinct lineage, paralleled with other genera within the family Anaerovoracaceae, suggesting that strain MT110^T^ represents a novel genus.

**Figure 2:**
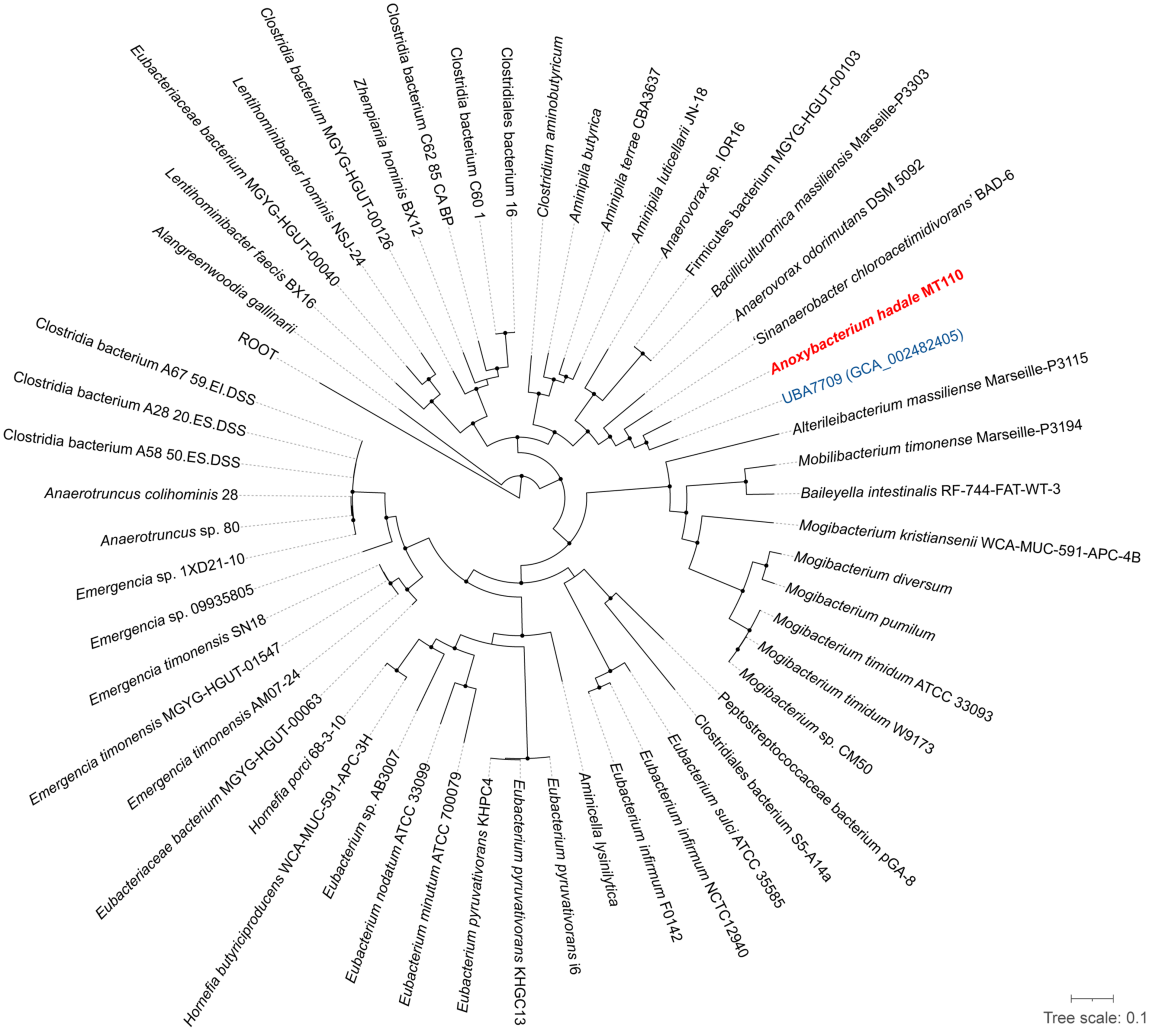
Phylogeny of strain MT110^T^ and the related species based on 120 concentration proteins. Strain MT110^T^ and a closely related metagenome-assembled genome (UBA7709) are highlighted in red and blue, respectively. The black nodes mean the bootstrap values ≥70%.

Furthermore, we have obtained the complete genome of MT110^T^, which consists of a circular chromosome of 4,941,017 bp with a G+C content of 44.6 mol% (Supplementary Figure S2). The genomic features of strain MT110^T^ are listed in Table 2. To determine the phylogenetic relationship of strain MT110^T^, a phylogenomic tree was constructed based on 120 conserved protein sequences (known as GTDB taxonomy). It revealed that strain MT110^T^ forms an independent monophyletic branch closely related to ‘UBA7709’ (metagenome-assembled genome GCA_002482405), followed by ‘*Sinanaerobacter chloroacetimidivorans*’ BAD-6 and *Anaerovorax odorimutans* DSM 5092^T^ (Figure 2). The POCP values between strains MT110^T^ and the closely related type strains, *Anaerovorax odorimutans* DSM 5092^T^ and *Aminipila butyrica* DSM 103574^T^, were 47.6 and 47.7%, respectively. The level of POCP is below the proposed 50% cut-off for the genus boundary of prokaryotic lineages (Qin et al., 2014).

**Table 2:** Genome features of strain MT110^T^.

Items	Description
Size (bp)	4,941,017
G+C content (%)	44.6
Coding sequence (%)	88.66
Total genes	4,330
Protein-coding genes	4,263
Genes assigned to COG	3,561
rRNA operons	4
tRNA genes	52
Gene islands	18
Non-coding RNA	3
CheckM2 completeness	100%
CheckM2 contamination	1.67%
Coverage	1,398-fold

Considering the NCBI and GTDB taxonomies, as well as the distinctive characteristics observed between strain MT110^T^ and neighboring genera, we propose that strain MT110^T^ belongs to a novel genus, *Anoxybacterium hadale* gen. nov. sp. nov., of the class Clostridia, which was previously uncultured.

### 3.3 Metabolic capabilities of strain MT110^T^

We discovered that strain MT110^T^ could grow strictly anaerobically in BM medium using peptone, tryptone, or yeast extract as a carbon source. However, when we exposed strain MT110^T^ to various substrates as the sole carbon source, such as sodium formate, sodium citrate, d-mannitol, *N*-acetyl-d-glucosamine, sodium fumarate, d-mannose, lactose, d-glucose, d-maltose, potassium d-gluconate, sodium pyruvate, l-arabinose, sodium lactate, d-fructose, sodium malate, d-galactose, sucrose, sodium acetate, sodium succinate, glycerol, 4-aminobutyric acid, sodium propionate, and sodium butyrate, none of them supported the growth of strain MT110^T^. We also explored this nutritional characteristic in several related strains, including *Aminipila butyrica* DSM 103574^T^ and *Anaerovorax odorimutans* DSM 5092^T^, and obtained similar results.

**Figure 3:**
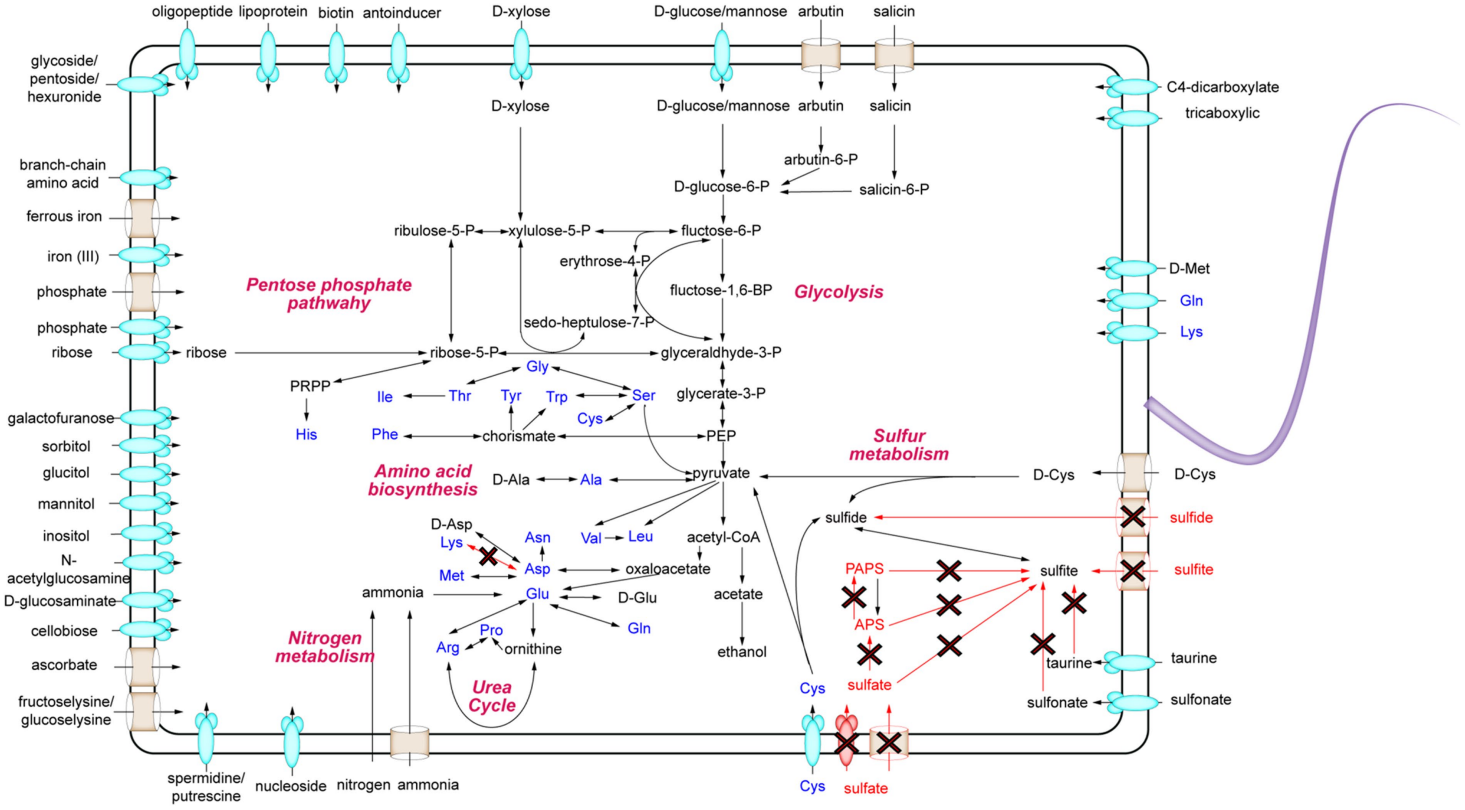
Schematic overview of the metabolic potential of strain MT110^T^. The red arrows indicate no path exists.

**Figure 4:**
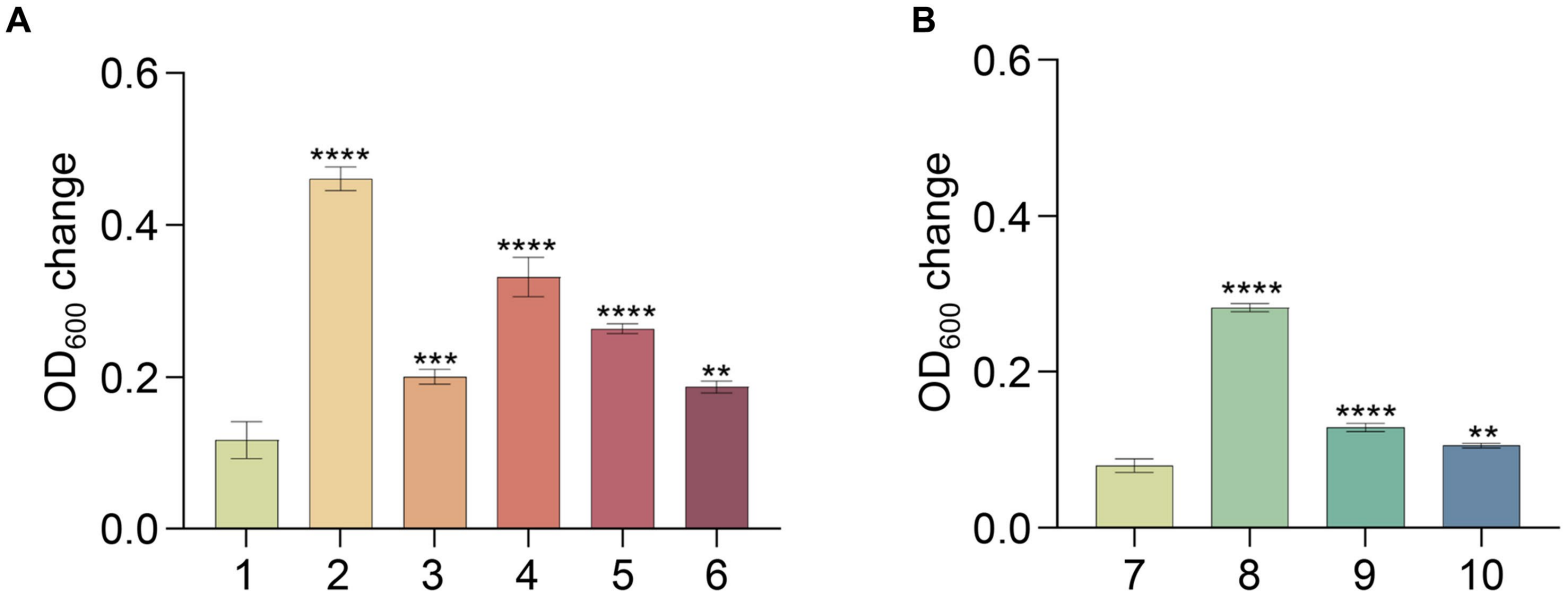
Effect of different substrates on growth of strain MT110^T^ in BMY medium. **(A)** and **(B)**, results of two independent experiments; 1 and 7, BYM control; 2, glucose; 3, xylose; 4, fructose; 5, inositol; 6, ribose; 8, sucrose; 9, cellobiose; 10, starch. *p ≤ 0.05, **p ≤ 0.01, ***p ≤ 0.001, ****p ≤ 0.0001.

To uncover the metabolic capabilities of strain MT110^T^, we reconstructed its metabolic pathways and identified its potential utilization of various carbon sources (Figure 3). Strain MT110^T^ possesses 338 genes involved in carbohydrate transport and metabolism (COG-G), which is more than other strains in the Anaerovoracaceae family (Supplementary Table S1). In fact, it harbors 107 genes associated with the transport of different carbohydrates, such as glucose, *N*-acetylglucosamine, cellobiose, ascorbate, ribose, mannitol, galactofuranose, d-glucosaminate, d-xylose, inositol, glucitol, sorbitol, fructoselysine/glucoselysine, and glycoside/pentoside/hexuronide (Supplementary Table S2). Notably, strain MT110^T^ shows a prediction of 10 ribose ABC transporter gene operons, indicating ribose as one of its primary carbon sources. Additionally, genes related to galactofuranose ABC transporter and fructoselysine/glucoselysine PTS system are uniquely present in strain MT110^T^ but absent in the other 51 strains of the Anaerovoracaceae family. Furthermore, we predict the presence of 22 glycoside hydrolase genes in strain MT110^T^, of which seven are extracellular enzymes fused with signal peptides, including xylan1,4-beta-xylosidase (GH43 family), chitinase (GH18 family), mannan endo-1,4-beta-mannosidase (GH26 family), and beta-*N*-acetylhexosaminidase (GH3 family) (Supplementary Table S3). These findings suggest that MT110^T^ may have the capability to degrade extracellular polysaccharides and oligosaccharides. To validate its carbohydrate utilization, we cultured strain MT110^T^ in BMY medium supplemented with various sugars such as glucose, xylose, fructose, ribose, inositol, sucrose, cellobiose, and water-soluble starch. The results showed a significantly enhanced growth of strain MT110^T^ in all the medium (Figure 4).

Furthermore, MT110^T^ predictively possess genes encoding malate permease, lactate permease, C4-dicarboxylate ABC transporters, and tricarboxylic ABC transporters (Supplementary Table S2). Additionally, MT110^T^ possesses a unique ascorbate PTS system, suggesting that carboxylic acids could also serve as a carbon source for MT110^T^.

For nitrogen metabolism, ammonium transporter, ethanolamine permease, spermidine/putrescine ABC transport system, adenine/guanine/hypoxanthine permease, xanthine permease, and general nucleoside transporter were predicted (Supplementary Table S2), suggesting that strain MT110^T^ could utilize various kinds of organic nitrogen sources. In addition, two operons of *nifHDKXB* and *nifH-nifHD1-nifHD2-nifDKEB-nuoF-nifV2-nifV* were predicted in strain MT110^T^, suggesting that MT110^T^ is also capable of obtaining ammonium through nitrogen fixation (Supplementary Table S4).

For phosphate and iron intake, the phosphate transport system (PtsABC), the PiT family inorganic phosphate transporter were identified. Additionally, polyphosphate kinase was predicted, suggesting that strain MT110^T^ could restore phosphate via the synthesis of polyphosphate. Moreover, all the genes of the iron (III) ABC transport system, ferrous iron transport proteins, and iron complex ABC transport system were found with at least two copy numbers in strain MT110^T^. These pieces of evidence suggest that carbon, nitrogen, phosphate, and iron may not be limiting factors for the growth of strain MT110^T^. This was further confirmed by our previous experiments, where a culture medium containing carbohydrates, ammonia, phosphate, and ferrous iron could not induce noticeable growth of strain MT110^T^.

### 3.4 The proteolytic characteristics of strain MT110^T^

Genome analysis showed that strain MT110^T^ has a total of 102 genes involved in the transport of amino acids and oligopeptides, including 5 gene operons for branched-chain amino acid ABC transporters and 4 operons for polar amino acid ABC transporters (Supplementary Table S2). Additionally, strain MT110^T^ is predicted to have several genes encoding extracellular (signalP-fused) S8/S53 family peptidases, M28 family peptidases, and oligoendopeptidase F family proteins.

Furthermore, several extracellular D-amyl peptidases were also predicted in MT110^T^, including zinc d-alanyl-d-alanine carboxypeptidase, serine-type d-Ala-d-Ala carboxypeptidase, d-methionine transport system, and d-cysteine desulfurase. These pieces of evidence suggest that the degradation of extracellular proteins and cell walls likely contributes to the survival of MT110^T^ in oligotrophic environments.

Considering that the genes involved in amino acid utilization are highly enriched in strain MT110^T^, we propose that genomic reduction has occurred in its genome, which could lead to the loss of certain amino acid synthetic genes and make it unable to grow using a single carbon source. Indeed, we found that the pathways of lysine synthesis from both 2,3,4,5-tetrahydrodipicolinate and 2-oxoadipic acid were largely incomplete. However, we still did not experimentally observe clear growth of strain MT110^T^ using the medium mixed with lysine and many kinds of carbohydrates as carbon sources.

### 3.5 Cysteine is essential for growth of strain MT110^T^

Finally, we focused on the sulfur metabolism of strain MT110^T^. Genomic comparison revealed the presence of sulfate reductase genes in ‘*Sinoanaerobacter chloroacetimidivorans*’ BAD-6 and *Anaerovorax odorimutans* DSM 5092^T^ but their absence in MT110^T^. Moreover, both sulfite and sulfide transporters were not found in MT110^T^. Additionally, although the genes of anaerobic sulfite reductase subunits (*asrABC*) were predicted in strain MT110^T^ (Supplementary Table S4), sulfite cannot exist in culture medium where sulfide was used as a deoxidizer. Conversely, we predicted several cysteine-degrading genes, including cysteine desulfurase (K04487), d-cysteine desulfurase (K05396), and cysteine desulfurase/selenocysteine lyase (K11717) in MT110^T^ (Supplementary Table S4). Based on these findings, we proposed that cysteine could be an essential nutrient for the growth of strain MT110^T^, where it may serve as a participant in protein synthesis and also provide S(−2) for other biochemical reactions.

**Figure 5:**
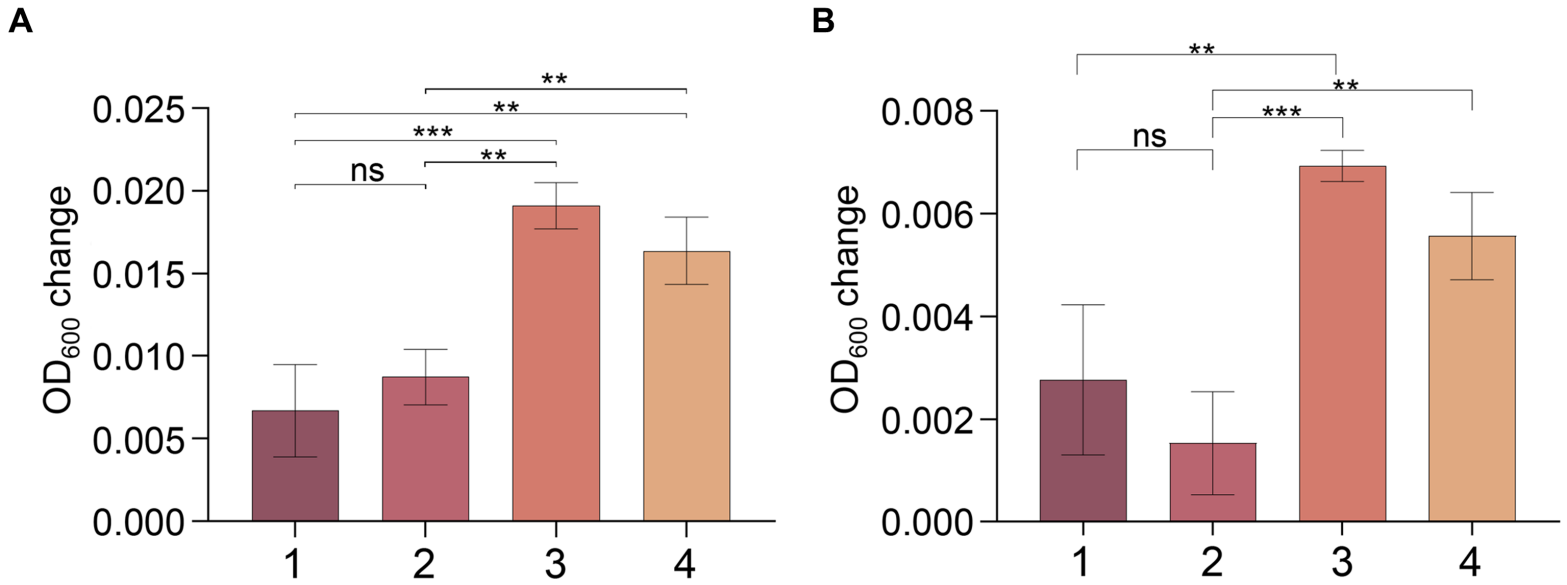
Effect of cysteine and methionine on growth of strain MT110^T^ in BM medium with 0.025% **(A)** and 0.05% **(B)** reducing agent (Na_2_S·9H_2_O). 1, 18 amino acids (except cysteine and methionine); 2, 18 amino acids and methionine; 3, 18 amino acids and cysteine; 4, 18 amino acids and methionine, cysteine. ^ns^*p* > 0.05, no significant correlation; *p ≤ 0.05, **p ≤ 0.01, ***p ≤ 0.001.

To confirm this hypothesis, we conducted an experiment with strain MT110^T^ using a medium consisting of 18 amino acids (excluding cysteine and methionine) as carbon and nitrogen sources, and sulfate and sulfide as sulfur sources. No clear signs of growth were observed for strain MT110^T^ under these conditions. However, when we supplemented the medium with 0.02% (w/v) cysteine (but not methionine), we detected obvious growth of strain MT110^T^, even after subsequent sulfate deprivation (Figure 5).

We also conducted an additional experiment where we reduced the amount of Na_2_S by half in the culture medium with mixed amino acids as carbon sources. Surprisingly, this reduction significantly increased the growth of strain MT110^T^ (Supplementary Figure S3). These findings confirm that sulfide, at least, cannot be utilized for cysteine synthesis in strain MT110^T^. In addition, we found that taurine and sulfonate were unable to support the growth of strain MT110^T^ in the absence of cysteine (data not shown), though transporter genes for them were predicted in the strain MT110^T^ genome. Taken together, these results indicate that cysteine plays a critical role in the fermentation process of strain MT110^T^.

## 4 Discussion

Deep-sea and hadal environments are typically considered to be organic carbon limited ecosystems, as most sinking particulate organic matter is either solubilized or mineralized by heterotrophic organisms before it reaches deep waters (Smith et al., 2008; Jamieson et al., 2010). Microbial growth on multi-carbon substrates may provide competitive advantages that allows microorganisms to relieve carbon limitation in the hadal environments, where the nutrient poor ‘food’ consist largely of refractory compounds. Although strain MT110^T^ could use none of the tested organic matter as sole carbon source, it does have the ability to degrade extracellular polysaccharides and oligosaccharides, such as glucose, xylose, fructose, ribose, inositol, sucrose, cellobiose, and water-soluble starch with the presence of yeast extract. These results demonstrate the novel isolate and related members may play an important role in the degradation of hadal organic matter and contribute to the biogeochemical cycle of carbon.

Sulfur, a component of amino acids, sulfolipids, and other biomolecules, is an essential nutrient for life and a crucial player in climate processes (Moran and Durham, 2019). Given that strain MT110^T^ did not assimilate inorganic sulfur, lacking organic sulfur nutrition seems to be the key factor making it genuinely resistant to growth with carbohydrate. This statement was evidenced by amino acids experiment, in which Cys was proved to be essential for its fermentation with protein-derived substates. This phenomenon was also observed in strain MT110^T^, *Anaerovorax* and *Aminipila* species, which are unable to reduce sulfate, sulfite or thiosulfate, but may take up organic sulfur, such as cysteine or methionine (Matthies et al., 2000; Wei et al., 2021). Therefore, organic sulfur compounds may play an important role in metabolism and growth of the family Anaerovoracaceae and maybe one of the key factors affecting the cultivation of the uncultured microbes. This statement could also elucidate some previously reported phenomenon, e.g., cultivation of *Abiotrophia* spp. (member of the family Aerococcaceae within the phylum Bacillota) and *Granulicatella* spp. (member of the family Carnobacteriaceae within the phylum Bacillota) requiring pyridoxal or cysteine (Vartoukian et al., 2010; Pham and Kim, 2012). Organic sulfur compounds in marine environments could support the growth of a diverse and specialized group of microorganisms and emerge as important ecological links between ocean microorganisms in the global carbon and sulfur cycles.

Interestingly, our result showed that methionine, another sulfur-containing amino acid, failed to support the growth of strain MT110^T^. It is probably due to its lacking cystathionine gamma-lyase (EC:4.4.1.1), which is a key enzyme transforming methionine to cysteine (Flavin and Segal, 1964). Although the genome contained genes encoding serine *O*-acetyltransferase (EC: 2.3.1.30) and cysteine synthase (EC: 2.5.1.47), which could transform serine and sulfide to cysteine, strain MT110^T^ still required cysteine for growth. This issue needs be addressed by further research in the future.

Cysteine, thiosulfate, sulfite, especially sulfide (S^2−^), are commonly used as reducing agents for anaerobic cultivation (Brock and Od'ea, 1977; Rothe and Thomm, 2000). In this study, we found that reducing the Na_2_S concentration could significantly increase the growth of MT110^T^ (Supplementary Figure S3), implying S2-may have a direct toxicity effect on anaerobic strain MT110^T^. Consequently, in the future, cysteine and thiosulfate could be used as reducing agent, and lowering the concentration of S2-would reduce the inhibition of anaerobic microbial growth.

## 5 Conclusion

The strictly anaerobic bacterial strain MT110^T^, isolated from a hadal sediment–water interface sample of the Mariana Trench, may represents a novel taxon at genus level and a previously uncultured lineage in the class Clostridia. Strain MT110^T^ could use none of the tested organic matter as sole carbon source but have the ability to degrade extracellular polysaccharides and oligosaccharides, such as glucose, xylose, fructose, ribose, inositol, sucrose, cellobiose, and water-soluble starch with the presence of yeast extract. Cysteine and other organic sulfur compounds play an important role in metabolism and growth of strain MT110^T^ and members of the family Anaerovoracaceae, and maybe one of the key factors affecting the cultivation of the uncultured hadal microbes. As such, the sulfur-containing amino acids, especially cysteine, may play an important role in the geochemical cycling of sulfur in hadal zones. In the future, genomic and experimental information obtained in the present study could be used to gain a deeper understanding of the trophic strategies of the family Anaerovoracaceae and relatives thriving in different environments. This work may also provide an additional clue in helping us culture not-yet-cultured microbes by mimicking the endogenous abiotic and biotic conditions required for microbial growth.

### 5.1 Description of *Anoxybacterium* gen. nov.

*Anoxybacterium* (An.oxy.bac.te′ri.um: Gr. pref. *an-*, without; M.L. *oxy*, shortened from oxygenium oxygen; L. neut. n. *bacterium*, rod; N.L. neut. n. *Anoxybacterium*, rod growing without oxygen).

Cells were Gram-positive, anaerobic, straight to gently curved rods with 1–3 flagella. The major fatty acids were C_14:0_, C_16:1_ *ω*7*c*/*ω*6*c* (Summed feature 3), C_16:0_ and C_18:1_ *ω*9*c*. The genomic DNA G+C content was 44.6 mol%.

The type species is *Anoxybacterium hadale*.

### 5.2 Description of *Anoxybacterium hadale* sp. nov.

*Anoxybacterium hadale* (ha.da′le. N.L. neut. Adj. *hadale* from Greek *Háidēs*, hadal of or relating to the deepest regions of the ocean).

Cells were Gram-positive, anaerobic, non-spore-forming, straight to gently curved rods (2.4–3.4 × 0.4–0.6 μm) with 1–3 flagella. Growth was observed at temperatures between 10 and 42°C (optimum 28°C), at NaCl concentration from 0 to 5% (optimum 0–0.5%) and at pH from 6 to 9 (optimum 7.5–8). The major fatty acids were C_14:0_ (47.7%), C_16:1_ *ω*7*c*/*ω*6*c* (Summed feature 3, 10.7%), C_16:0_ (10.3%) and C_18:1_ *ω*9*c* (10.0%). The genomic DNA G+C content was 44.6 mol%.

The type strain, MT110^T^ (=KCTC 15922^T^ = MCCC 1K04061^T^), was isolated from a hadal sediment–water interface sample collected at a depth of 10,890 m from Mariana Trench (11.4° N, 142.4° E, site MT).

## Data Availability

The datasets presented in this study can be found in online repositories. The names of the repository/repositories and accession number(s) can be found at: https://www.ncbi.nlm.nih.gov/genbank/, OQ442995 and CP042469.

## Footnotes

^1^https://lpsn.dsmz.de, accessed on July 17, 2024.

^2^ https://www.ezbiocloud.net/identify

## References

[ref1] Aromokeye D. A. Oni O. E. Tebben J. Yin X. Friedrich M. W. (2020). Crystalline iron oxides stimulate methanogenic benzoate degradation in marine sediment-derived enrichment cultures. ISME J. 15, 965–980. doi: 10.1038/s41396-020-00824-7 33154547 PMC8115662

[ref2] Balch W. E. Fox G. E. Magrum L. J. Woese C. R. Wolfe R. S. (1979). Methanogens: reevaluation of a unique biological group. Microbiol. Rev. 43, 260–296. doi: 10.1128/mr.43.2.260-296.1979, PMID: 390357 PMC281474

[ref3] Bertelli C. Laird M. R. Williams K. P. Simon Fraser University Research Computing, G Lau B. Y. Hoad G.. (2017). IslandViewer 4: expanded prediction of genomic islands for larger-scale datasets. Nucleic Acids Res. 45, W30–W35. doi: 10.1093/nar/gkx343 28472413 PMC5570257

[ref4] Brock T. D. Od'ea K. (1977). Amorphous ferrous sulfide as a reducing agent for culture of anaerobes. Appl. Environ. Microbiol. 33, 254–256. doi: 10.1128/aem.33.2.254-256.1977, PMID: 192144 PMC170674

[ref5] Cao J. Gayet N. Zeng X. Shao Z. Jebbar M. Alain K. (2016). *Pseudodesulfovibrio indicus* gen. nov., sp. nov., a piezophilic sulfate-reducing bacterium from the Indian Ocean and reclassification of four species of the genus *Desulfovibrio*. Int. J. Syst. Evol. Microbiol. 66, 3904–3911. doi: 10.1099/ijsem.0.001286, PMID: 27392787

[ref6] Chaumeil P. A. Mussig A. J. Hugenholtz P. Parks D. H. (2019). GTDB-Tk: a toolkit to classify genomes with the genome taxonomy database. Bioinformatics. 36, 1925–1927. doi: 10.1093/bioinformatics/btz848 31730192 PMC7703759

[ref7] Chklovski A. Parks D. H. Woodcroft B. J. Tyson G. W. (2023). CheckM2: a rapid, scalable and accurate tool for assessing microbial genome quality using machine learning. Nat. Methods 20, 1203–1212. doi: 10.1038/s41592-023-01940-w, PMID: 37500759

[ref8] Danovaro R. Gambi C. Della Croce N. (2002). Meiofauna hotspot in the Atacama trench, eastern South Pacific Ocean. Deep-Sea Res. I Oceanogr. Res. Pap. 49, 843–857. doi: 10.1016/S0967-0637(01)00084-X

[ref9] Elbourne L. D. Tetu S. G. Hassan K. A. Paulsen I. T. (2017). TransportDB 2.0: a database for exploring membrane transporters in sequenced genomes from all domains of life. Nucleic Acids Res. 45, D320–D324. doi: 10.1093/nar/gkw1068, PMID: 27899676 PMC5210551

[ref10] Felsenstein J. (1981). Evolutionary trees from DNA sequences: a maximum likelihood approach. J. Mol. Evol. 17, 368–376. doi: 10.1007/BF01734359 7288891

[ref11] Flavin M. Segal A. (1964). Purification and properties of the cystathionine *γ*-cleavage enzyme of *Neurospora*. J. Biol. Chem. 239, 2220–2227. doi: 10.1016/S0021-9258(20)82223-6, PMID: 14209951

[ref12] Glud R. N. (2008). Oxygen dynamics of marine sediments. Mar. Biol. Res. 4, 243–289. doi: 10.1080/17451000801888726

[ref13] Guan H. Chen L. Luo M. Liu L. Mao S. Ge H.. (2019). Composition and origin of lipid biomarkers in the surface sediments from the southern challenger deep, Mariana trench. Geosci. Front. 10, 351–360. doi: 10.1016/j.gsf.2018.01.004

[ref14] Hu X. Liu J. Liu H. Zhuang G. Xun L. (2018). Sulfur metabolism by marine heterotrophic bacteria involved in sulfur cycling in the ocean. Sci. China Earth Sci. 61, 1369–1378. doi: 10.1007/s11430-017-9234-x

[ref15] Hyunwoo L. Fitamo T. M. Nesb C. L. Guilford N. G. H. Krt K. Ivy Y. M.. (2023). Microbial community dynamics of a sequentially fed anaerobic digester treating solid organic waste. FEMS Microbiol. Ecol. 3:fiad017. doi: 10.1093/femsec/fiad017 36809778

[ref16] Jamieson A. J. Fujii T. Mayor D. J. Solan M. Priede I. G. (2010). Hadal trenches: the ecology of the deepest places on earth. Trends Ecol. Evol. 25, 190–197. doi: 10.1016/j.tree.2009.09.009, PMID: 19846236

[ref17] Johnson J. S. Spakowicz D. J. Hong B. Y. Petersen L. M. Demkowicz P. Chen L.. (2019). Evaluation of 16S rRNA gene sequencing for species and strain-level microbiome analysis. Nat. Commun. 10:5029. doi: 10.1038/s41467-019-13036-1 31695033 PMC6834636

[ref18] Kanehisa M. Sato Y. Morishima K. (2016). BlastKOALA and GhostKOALA: KEGG tools for functional characterization of genome and metagenome sequences. J. Mol. Biol. 428, 726–731. doi: 10.1016/j.jmb.2015.11.006, PMID: 26585406

[ref19] Kim Y. B. Kim J. Y. Kim J. Song H. S. Whon T. W. Lee S. H.. (2021). *Aminipila terrae* sp. nov., a strictly anaerobic bacterium isolated from river sediment. Arch. Microbiol. 203, 3163–3169. doi: 10.1007/s00203-021-02301-x 33821299

[ref20] Kim W. Lee J.-H. Kwon K. K. (2016). Isolation and characterization of anaerobic microbes from marine environments in Korea. Korean J. Microbiol. 52, 183–191. doi: 10.7845/kjm.2016.6010

[ref21] Kobayashi H. Saito N. Fu Q. Kawaguchi H. Vilcaez J. Wakayama T.. (2013). Bio-electrochemical property and phylogenetic diversity of microbial communities associated with bioelectrodes of an electromethanogenic reactor. J. Biosci. Bioeng. 116, 114–117. doi: 10.1016/j.jbiosc.2013.01.001, PMID: 23415665

[ref22] Koren S. Walenz B. P. Berlin K. Miller J. R. Bergman N. H. Phillippy A. M. (2017). Canu: scalable and accurate long-read assembly via adaptive k-mer weighting and repeat separation. Genome Res. 27, 722–736. doi: 10.1101/gr.215087.116, PMID: 28298431 PMC5411767

[ref23] Kumar S. Stecher G. Li M. Knyaz C. Tamura K. (2018). MEGA X: molecular evolutionary genetics analysis across computing platforms. Mol. Biol. Evol. 35, 1547–1549. doi: 10.1093/molbev/msy096, PMID: 29722887 PMC5967553

[ref24] Letunic I. Bork P. (2021). Interactive tree of life (iTOL) v5: an online tool for phylogenetic tree display and annotation. Nucleic Acids Res. 49, W293–W296. doi: 10.1093/nar/gkab301, PMID: 33885785 PMC8265157

[ref25] Li Y. Cao W. Wang Y. Ma Q. (2019). Microbial diversity in the sediments of the southern Mariana trench. J. Oceanol. Limnol. 37, 1024–1029. doi: 10.1007/s00343-019-8131-z

[ref26] Li L. Stoeckert C. J. Jr. Roos D. S. (2003). OrthoMCL: identification of ortholog groups for eukaryotic genomes. Genome Res. 13, 2178–2189. doi: 10.1101/gr.1224503, PMID: 12952885 PMC403725

[ref27] Li Z. Suzuki D. Zhang C. Yang S. Nan J. Yoshida N.. (2014). Anaerobic 4-chlorophenol mineralization in an enriched culture under iron-reducing conditions. J. Biosci. Bioeng. 118, 529–532. doi: 10.1016/j.jbiosc.2014.04.007, PMID: 24794625

[ref28] Liu Y. Chen S. Wang J. Shao B. Fang J. Cao J. (2023). The phylogeny, metabolic potentials, and environmental adaptation of an anaerobe, *Abyssisolibacter* sp. M8S5, isolated from cold seep sediments of the South China Sea. Microorganisms 11:2156. doi: 10.3390/microorganisms11092156 37764000 PMC10536192

[ref29] Liu P. Ding W. Lai Q. Liu R. Wei Y. Wang L.. (2020). Physiological and genomic features of *Paraoceanicella profunda* gen. nov., sp. nov., a novel piezophile isolated from deep seawater of the Mariana trench. Microbiol. Open 9:e966. doi: 10.1002/mbo3.966, PMID: 31743595 PMC7002103

[ref30] Liu R. Wang L. Wei Y. Fang J. (2018). The hadal biosphere: recent insights and new directions. Deep-Sea Res. II Top. Stud. Oceanogr. 155, 11–18. doi: 10.1016/j.dsr2.2017.04.015

[ref31] Liu J. Wu W. Chen C. Sun F. Chen Y. (2011). Prokaryotic diversity, composition structure, and phylogenetic analysis of microbial communities in leachate sediment ecosystems. Appl. Microbiol. Biotechnol. 91, 1659–1675. doi: 10.1007/s00253-011-3354-8, PMID: 21637937

[ref32] Loman N. J. Quick J. Simpson J. T. (2015). A complete bacterial genome assembled de novo using only nanopore sequencing data. Nat. Methods 12, 733–735. doi: 10.1038/nmeth.3444, PMID: 26076426

[ref33] Matthies C. Evers S. Ludwig W. Schink B. (2000). *Anaerovorax odorimutans* gen. nov., sp. nov., a putrescine-fermenting, strictly anaerobic bacterium. Int. J. Syst. Evol. Microbiol. 50, 1591–1594. doi: 10.1099/00207713-50-4-1591 10939665

[ref34] Moran M. A. Durham B. P. (2019). Sulfur metabolites in the pelagic ocean. Nat. Rev. Microbiol. 17, 665–678. doi: 10.1038/s41579-019-0250-1, PMID: 31485034

[ref35] Neupane S. Ghosh A. Gunther S. Martin K. Zurek L. (2020). *Culicoidibacter larvae* gen. nov., sp. nov., from the gastrointestinal tract of the biting midge (*Culicoides sonorensis*) larva, belongs to a novel lineage *Culicoidibacteraceae* fam. nov., *Culicoidibacterales* ord. nov. and *Culicoidibacteria classis* nov. of the phylum Firmicutes. Int. J. Syst. Evol. Microbiol. 70, 6482–6490. doi: 10.1099/ijsem.0.004543, PMID: 33125314

[ref36] Nunoura T. Takaki Y. Hirai M. Shimamura S. Makabe A. Koide O.. (2015). Hadal biosphere: insight into the microbial ecosystem in the deepest ocean on earth. Proc. Natl. Acad. Sci. USA 112, E1230–E1236. doi: 10.1073/pnas.1421816112, PMID: 25713387 PMC4371994

[ref37] Pham V. H. Kim J. (2012). Cultivation of unculturable soil bacteria. Trends Biotechnol. 30, 475–484. doi: 10.1016/j.tibtech.2012.05.007 22770837

[ref38] Price M. N. Dehal P. S. Arkin A. P. (2010). FastTree 2 – approximately maximum-likelihood trees for large alignments. PLoS One 5:e9490. doi: 10.1371/journal.pone.0009490, PMID: 20224823 PMC2835736

[ref39] Qin W. (2016). Recent developments in using advanced sequencing technologies for the genomic studies of lignin and cellulose degrading microorganisms. Int. J. Biol. Sci. 12:156. doi: 10.7150/ijbs.13537 26884714 PMC4737673

[ref40] Qin Q. L. Xie B. B. Zhang X. Y. Chen X. L. Zhou B. C. Zhou J.. (2014). A proposed genus boundary for the prokaryotes based on genomic insights. J. Bacteriol. 196, 2210–2215. doi: 10.1128/JB.01688-14, PMID: 24706738 PMC4054180

[ref41] Rothe O. Thomm M. (2000). A simplified method for the cultivation of extreme anaerobic archaea based on the use of sodium sulfite as reducing agent. Extremophiles 4, 247–252. doi: 10.1007/PL00010716, PMID: 10972193

[ref42] Sasser M. (1990). Identification of bacteria by gas chromatography of cellular fatty acids. Newark, DE: MIDI.

[ref43] Sievers F. Higgins D. G. (2021). The Clustal omega multiple alignment package. Methods Mol. Biol. 2231, 3–16. doi: 10.1007/978-1-0716-1036-7_1, PMID: 33289883

[ref44] Smith C. R. De Leo F. C. Bernardino A. F. Sweetman A. K. Arbizu P. M. (2008). Abyssal food limitation, ecosystem structure and climate change. Trends Ecol. Evol. 23, 518–528. doi: 10.1016/j.tree.2008.05.002, PMID: 18584909

[ref45] Staudinger T. Pipal A. Redl B. (2011). Molecular analysis of the prevalent microbiota of human male and female forehead skin compared to forearm skin and the influence of make-up. J. Appl. Microbiol. 110, 1381–1389. doi: 10.1111/j.1365-2672.2011.04991.x 21362117

[ref46] Tatusov R. L. Natale D. A. Garkavtsev I. V. Tatusova T. A. Shankavaram U. T. Rao B. S.. (2001). The COG database: new developments in phylogenetic classification of proteins from complete genomes. Nucleic Acids Res. 29, 22–28. doi: 10.1093/nar/29.1.22, PMID: 11125040 PMC29819

[ref47] Tatusova T. Dicuccio M. Badretdin A. Chetvernin V. Nawrocki E. P. Zaslavsky L.. (2016). NCBI prokaryotic genome annotation pipeline. Nucleic Acids Res. 44, 6614–6624. doi: 10.1093/nar/gkw569, PMID: 27342282 PMC5001611

[ref48] Tischer K. Kleinsteuber S. Schleinitz K. M. Fetzer I. Spott O. Stange F.. (2013). Microbial communities along biogeochemical gradients in a hydrocarbon-contaminated aquifer. Environ. Microbiol. 15, 2603–2615. doi: 10.1111/1462-2920.12168, PMID: 23809669

[ref49] Ueki A. Goto K. Kaku N. Ueki K. (2018). *Aminipila butyrica* gen. Nov., sp. nov., a strictly anaerobic, arginine-decomposing bacterium isolated from a methanogenic reactor of cattle waste. Int. J. Syst. Evol. Microbiol. 68, 443–448. doi: 10.1099/ijsem.0.002534, PMID: 29235979

[ref50] Vartoukian S. R. Palmer R. M. Wade W. G. (2010). Strategies for culture of ‘unculturable’ bacteria. FEMS Microbiol. Lett. 309, 1–7. doi: 10.1111/j.1574-6968.2010.02000.x 20487025

[ref51] Wei Z. Ma S. Chen R. Wu W. Fan H. Dai L.. (2021). *Aminipila luticellarii* sp. nov., an anaerobic bacterium isolated from the pit mud of strong aromatic Chinese liquor, and emended description of the genus *Aminipila*. Int. J. Syst. Evol. Microbiol. 71:004710. doi: 10.1099/ijsem.0.004710 34662267

[ref52] Widdel F. Bak F. (1992). “Gram-negative mesotrophic sulfate-reducing bacteria” in The prokaryotes. eds. Balows A. Trüper H. G. Dworkin M. Harder W. Schleifer K. (New York, NY: Springer).

[ref53] Yoon S. H. Ha S. M. Kwon S. Lim J. Kim Y. Seo H.. (2017). Introducing EzBioCloud: a taxonomically united database of 16S rRNA gene sequences and whole-genome assemblies. Int. J. Syst. Evol. Microbiol. 67, 1613–1617. doi: 10.1099/ijsem.0.001755 28005526 PMC5563544

