# Supplementary material for: Genomic and physiological properties of Anoxybacterium hadale gen. nov. sp. nov. reveal the important role of dissolved organic sulfur in microbial metabolism in hadal ecosystems
Source: Front Microbiol. 2024 Aug 16;15:1423245. doi: 10.3389/fmicb.2024.1423245 (PMC11362086; doi:10.3389/fmicb.2024.1423245)
Supplement: Supplementary file 5 [file Data_Sheet_1.pdf]

Genomic and physiological properties of *Anoxybacterium hadale* gen. nov. sp. nov. reveal the important role of dissolved organic sulfur in microbial metabolism in hadal ecosystems

Junwei Cao[1,†], Baoying Shao[1,†], Jing Lin[1], Jie Liu[1], Yiran Cui[1], Jiahua Wang[1,*] and Jiasong Fang[1,2,*]

[1]College of Oceanography and Ecological Science, Shanghai Ocean University, Shanghai, China

[2]Laboratory for Marine Biology and Biotechnology, Qingdao National Laboratory for Marine Science and Technology, Qingdao, China

[*] **Correspondence:**

Jiahua Wang (sjtu_wangjiahua@163.com) and Jiasong Fang (jsfang@shou.edu.cn)

[†]These authors contributed equally to this work.

Supplementary material

Supplementary Table 1: The COG categories of strain MT110^T and neighboring strains.

Supplementary Table 2: The transporter genes in strain MT110^T.

Supplementary Table 3: The glycoside hydrolase coding genes in strain MT110^T.

Supplementary Table 4: Genes involved in nitrogen fixation, cysteine degradation and sulfite reduction in strain MT110^T.

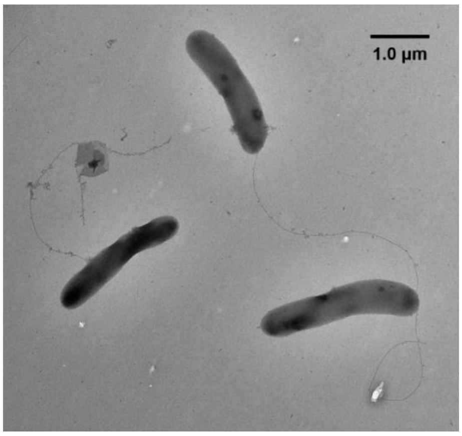

Supplementary Figure 1. Transmission electron microscopy image of cell of strain MT110^T grown on M89 medium at 28 °C for 90 h.

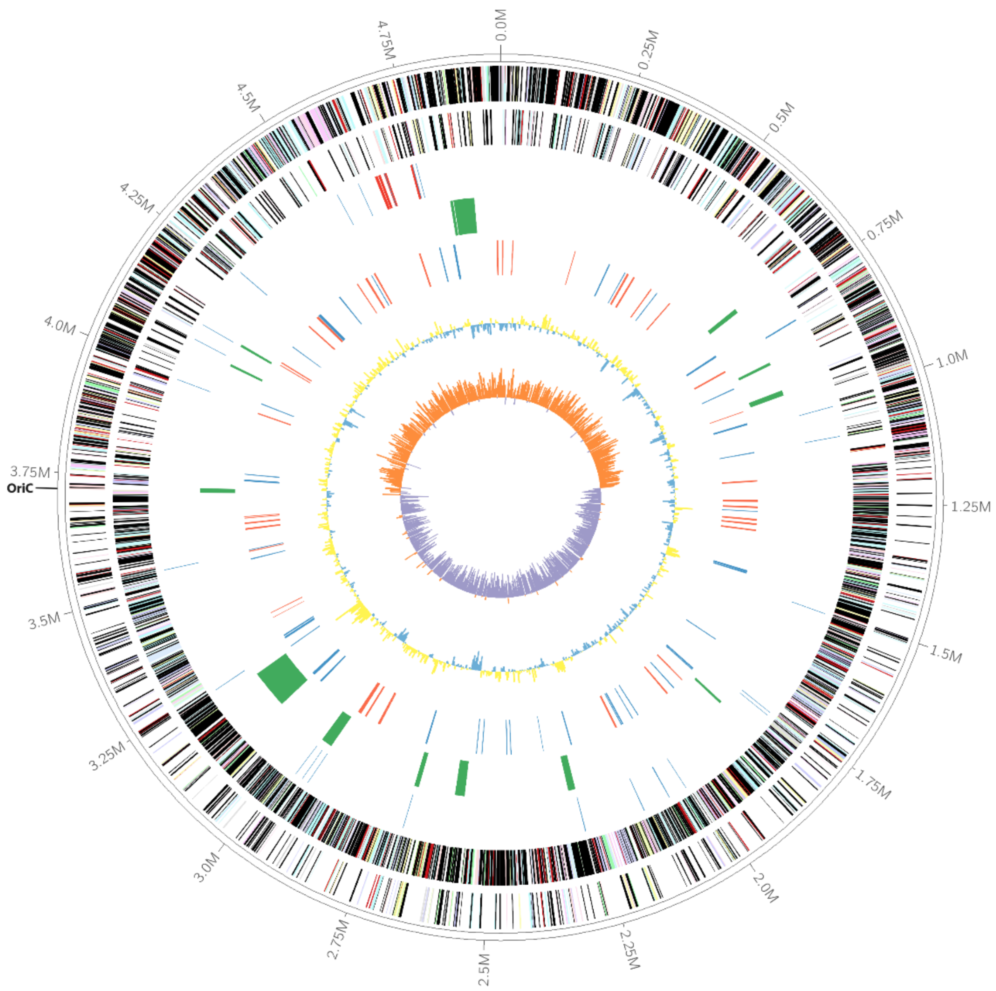

Supplementary Figure 2. Graphical representation of the MT110^{T} genome. Genes on the forward (shown in the outer circle) and reverse (shown in the inner circle) strands are colored according to their cluster of orthologous gene (COG) categories (except those colored in black for no hits); RNA genes are highlighted with different colors (tRNAs blue and rRNAs red); gene islands are shown in green; genes of transposases, recombinases, and integrases are shown in orange; proviruses are show in different colors (red for high qualified, pink for medium qualified, and blue for low qualified); GC content is shown in yellow/blue; and GC skew is shown in orange/purple (window, 5000 bp; step, 2500 bp).

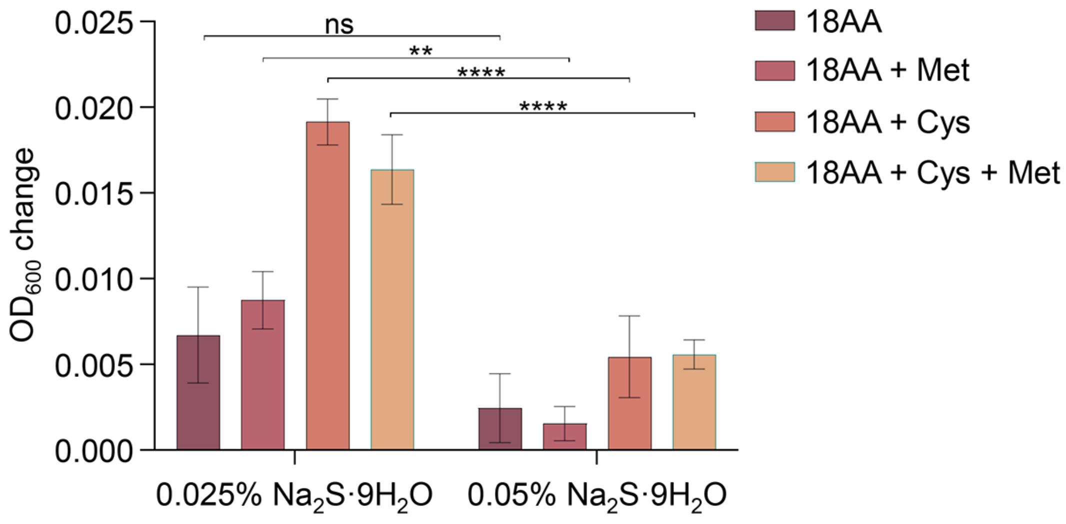

Supplementary Figure 3. Effect of different deaerating agent concentration on growth of MT110^T. ns, $p > 0.05$, no significant correlation; *, p≤0.05; **, p≤0.01; ***, p≤0.001; ****, p≤0.0001.
